# EVI1 phosphorylation at S436 regulates interactions with CtBP1 and DNMT3A and promotes self-renewal

**DOI:** 10.1038/s41419-020-03099-0

**Published:** 2020-10-20

**Authors:** Roberto Paredes, James R. Kelly, Bethany Geary, Batool Almarzouq, Marion Schneider, Stella Pearson, Prakrithi Narayanan, Andrew Williamson, Simon C. Lovell, Daniel H. Wiseman, John A. Chadwick, Nigel J. Jones, Olga Kustikova, Axel Schambach, Terence Garner, Fabio M. R. Amaral, Andrew Pierce, Adam Stevens, Tim C. P. Somervaille, Anthony D. Whetton, Stefan Meyer

**Affiliations:** 1 Stem Cell and Leukaemia Proteomics Laboratory, Division of Cancer Sciences, Faculty of Biology, Medicine and Health, University of Manchester, Manchester, UK; 2 Manchester Academic Health Science Centre, National Institute for Health Research Biomedical Research Centre, Manchester, UK; 3 Department of Biochemistry, Institute of Integrative Biology/School of Life Sciences, University of Liverpool, Liverpool, UK; 4 Division of Evolution and Genomic Sciences, School of Biological Sciences, University of Manchester, Manchester, UK; 5 Epigenetics of Haematopoiesis Laboratory, Division of Cancer Sciences, The University of Manchester, Manchester, UK; 6 Leukaemia Biology Laboratory, CRUK Manchester Institute, The University of Manchester, Manchester, UK; 7 Institute of Experimental Hematology, Hannover Medical School, Hannover, Germany; 8 Division of Developmental Biology and Medicine, Faculty of Biology, Medicine and Health, University of Manchester, Manchester, UK; 9 Stoller Biomarker Discovery Centre, University of Manchester, Manchester, UK; 10 Department of Paediatric Haematology and Oncology, Royal Manchester Children’s Hospital, Manchester, UK; 11 Young Oncology Unit, The Christie NHS Foundation Trust, Manchester, UK

**Keywords:** Acute myeloid leukaemia, Leukaemia

## Abstract

The transcriptional regulator EVI1 has an essential role in early development and haematopoiesis. However, acute myeloid leukaemia (AML) driven by aberrantly high *EVI1* expression has very poor prognosis. To investigate the effects of post-translational modifications on EVI1 function, we carried out a mass spectrometry (MS) analysis of EVI1 in AML and detected dynamic phosphorylation at serine 436 (S436). Wild-type EVI1 (EVI1-WT) with S436 available for phosphorylation, but not non-phosphorylatable EVI1-S436A, conferred haematopoietic progenitor cell self-renewal and was associated with significantly higher organised transcriptional patterns. In silico modelling of EVI1-S436 phosphorylation showed reduced affinity to CtBP1, and CtBP1 showed reduced interaction with EVI1-WT compared with EVI1-S436A. The motif harbouring S436 is a target of CDK2 and CDK3 kinases, which interacted with EVI1-WT. The methyltransferase DNMT3A bound preferentially to EVI1-WT compared with EVI1-S436A, and a hypomethylated cell population associated by EVI1-WT expression in murine haematopoietic progenitors is not maintained with EVI1-S436A. These data point to EVI1-S436 phosphorylation directing functional protein interactions for haematopoietic self-renewal. Targeting EVI1-S436 phosphorylation may be of therapeutic benefit when treating EVI1-driven leukaemia.

## Introduction

**Fig. 1:**
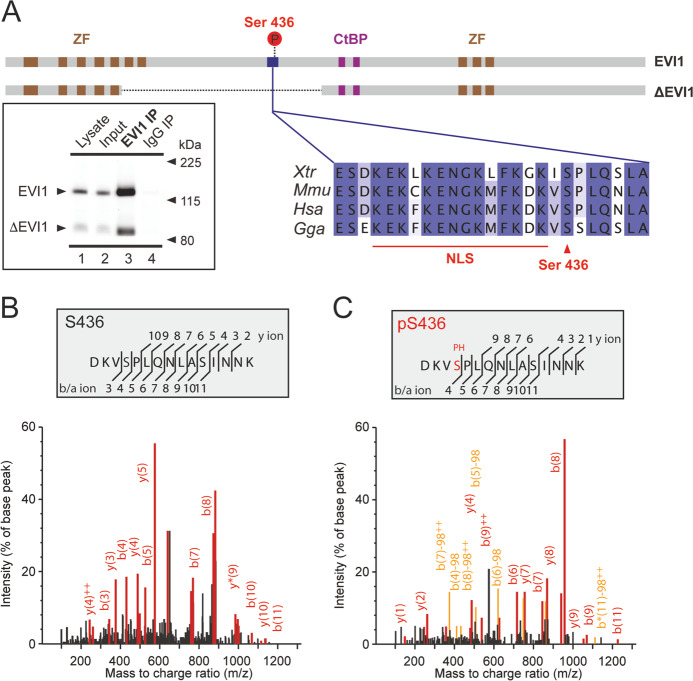
EVI1 is phosphorylated at S436. **a** Schematic illustration of the EVI1 and ΔEVI1-protein isoforms showing DNA binding zinc finger domains (ZF) and CtBP-binding motifs (CtBP). S436 phosphorylation shown in a red circle, in relation to the EVI1-SPLQ motif EVI1 with other species and the putative nuclear localization signal (NLS). Immunoprecipitation of endogenously expressed EVI1 is from SB1690CB cells shown in the western blot of EVI1. **b, c** Mass spectrometry analysis of the EVI1 peptide Asp433-Lys447 from SB1690CB AML cells non-phosphorylated (**b**) and S436-phosphorylated peptides (**c**).

EVI1 is a transcriptional regulator with essential functions in early haematopoiesis and development^1,2^. Aberrantly high expression of *EVI1*, often caused by chromosomal rearrangements involving the *MECOM* (MDS-EVI1 complex) locus at 3q26, where *EVI1* is encoded, is highly oncogenic. Acute myeloid leukaemia (AML) with high *EVI1* expression is one of the most aggressive forms of AML with poor outcome^3,4^. How overexpressed *EVI1* drives transformation to chemo-resistant leukaemia is incompletely understood. Several protein isoforms are transcribed from the *MECOM* locus, of which the 1051 amino acid (aa) EVI1 isoform confers most oncogenic properties when aberrantly expressed at high level^5,6^. This EVI1 isoform consists of an N-terminal zinc finger domain with seven motifs, a proline-rich central repressor domain, a smaller C-terminal zinc finger domain with three motifs, and a carboxy-terminal acidic domain (Fig. 1a). A longer MDS-EVI1 isoform, which has an additional N-terminal PR domain with methyltransferase activity, supports normal haematopoietic self-renewal, and can be expressed alongside the other EVI1-isoforms^7^. The shorter ΔEVI1 isoform, which lacks 342 aa (190–514) including the seventh zinc finger of the N-terminal zinc finger domain, is ordinarily co-expressed with EVI1 (Fig. 1a). ΔEVI1 does not sustain normal development in mice and cannot transform Rat-1 fibroblasts^8,9^. This implies an essential function of the sequence outspliced in ΔEVI1. Clinical detection of the heterozygous mutation c.1302_1306del in this region in an infant with bone marrow failure further supports its essential role in normal haematopoiesis^10^. EVI1 modulates gene expression by direct binding to specific DNA sequences^11,12^, and engages in dynamic interactions with other transcriptionally active proteins and epigenetic regulators^6,13,14^. EVI1 interactions are in part governed by post-translational modifications^13,15^. To further understand the role of individual sites of EVI1 phosphorylation we have analysed endogenously expressed EVI1 from AML cells. We report here on the phosphorylation at EVI1 serine 436 (S436), which is located in a functionally essential region of EVI1. We provide evidence and mechanistic insights for a role of this phosphorylation in EVI1-mediated haematopoietic self-renewal.

## Materials and methods

### Cell lines and tissue culture

The EVI1 expressing AML cell line SB1690CB, the EVI1-negative AML cell line OCI-AML5, HEK293T cells and Rat-1 fibroblasts were cultured as previously described^15,16^. All cell lines were regularly authenticated by STR profiling and were mycoplasma free. Murine haematopoietic progenitor cells were isolated, purified and maintained as described previously^15^. For further details, see Supplementary Materials and Methods.

### Antibodies

For detection and immunoprecipitation of human EVI1 a polyclonal antibody raised against the N-terminal EVI1 epitope MKSEDYPHETMAPDI (Eurogentec, Seraing, Belgium), and the EVI1 antibodies #2265 and #2593 (Cell Signaling Technology (CST), Leiden, The Netherlands) were used. For other antibodies, see Supplementary Materials and Methods, Supplementary Table 1.

### Immunoprecipitation, co-immunoprecipitation and western blot analysis

Cell lysis and immunoprecipitation were carried out as previously described^15,16^. EVI1 was immunoprecipitated with EVI1 antibody #2593 (CST) and captured with protein A sepharose beads. For immunoprecipitation of flag-tagged proteins transfected cells were incubated for one hour with FlagM2 magnetic beads (Sigma, Darmstadt, Germany). Protein electrophoresis and western blots were carried out using standard methodologies. For western blot quantification see Supplementary Materials and Methods.

### Plasmids and site-directed mutagenesis

The human EVI1 coding region was excised from pBABE-puro-flag-EVI1 (gift from Aubrey Thompson) using *Sal*I and *EcoR*I restriction sites and inserted into the *Sal*I and *EcoR*I sites of pCMV-flag-5a. Substitution of S436 with alanine (A) to create the vector pCMV-EVI1-S436A-flag was done by site-directed mutagenesis using the QuikChange^®^ II XL Kit (Agilent, Cheadle, UK). The lentiviral vector expressing codon-optimized mouse *Evi1*- pRRL.PPT.SF.EVI1mCo.IRES_EGFP.pre was mutated as above to generate pRRL.PPT.SF.EVI1mCoS436.IRES_EGFP.pre^15,17–19^. Control pRRL.PPT.SF.IRES_EGFP.pre was generated by excision of the Evi1-ORF from pRRL.PPT.SF.Evi1mCo.IRES_EGFP.pre vector with Bam*H*I restriction enzyme and religated to create an empty backbone vector. Lentiviral packaging vectors pHCMV-G, pMDLg/pRRE and pRSV-Rev were used as described. Primer sequences are provided in the Supplementary Material Table 2. Confirmation of mutated sequence is illustrated in Supplementary Fig. 1. Tagged DNMT3A was expressed using the cDNA-HA-DNMT3A plasmid encoding HA-tagged DNMT3A^20^.

### Reporter gene analysis

Reporter gene assays were carried out as described before^15^ (details in Supplementary Materials and Methods) with western-blot-monitored EVI1 expression (Supplementary Fig. 1b).

### Serial replating of haematopoietic progenitors

Murine haematopoietic c-Kit^+^ stem and progenitor cells (KIT^+^ HSPCs) were isolated from bone marrow of 8–10-week-old C57/BL6 mice as previously described^15^. Lentiviral-mediated transduction was carried out with the EVI1 vectors pRRL.PPT.SF.EVI1mCo.IRES_EGFP.pre, and site mutated pRRL.PPT.SF.EVI1mCoS436.IRES_EGFP.pre as described. In brief, cells cultured in medium supplemented with 4 μg/mL protamine (Sigma) were sequentially infected with two viral batches by spinoculation (60 min at 1250 × *g* at 32 °C), and cultured for two days in prestimulation serum-free cytokine supplemented medium (StemCell Technologies, Cambridge, UK), prior to FACS selection of GFP^+^ cells. Equal transduction efficiency for Evi1-WT and Evi1-S436A was monitored by RT-PCR and quantitation of fluorescent signal (Supplementary Fig. 1). For replating 2 × 10^4^ GFP^+^ cells were plated in cytokine supplemented Methocult M3231 medium. After 7 days in culture, colonies were counted and morphologically assessed. Cells were then harvested, and 2 × 10^4^ cells were replated as before. Colonies were scored for subsequent rounds of replating as above. For morphological analysis cytospin preparations were stained with May–Grünwald Giemsa (Supplementary Fig. 1), and 200 cells per preparation were assessed.

### Immunofluorescence (IF)

For immunofluorescence cells were incubated with antibodies following standard procedures and image generation (further details in Supplementary Materials and Methods).

Co-localisation of EVI1 and 5 mC signals was assessed by determination of Pearson correlation coefficient ranging from +1 (maximal correlation) to −1 (maximal anti-correlation)*. r* and *p* values were calculated using the free source Social Science Statistics.

### Gene expression analysis

For Poly-A RNA sequencing (RNA*seq*) analysis of Evi1-mediated modulation of gene expression RNA was extracted from murine haematopoietic c-Kit^+^ stem and progenitor cells (KIT^+^ HSPCs) at 48 h after lentiviral transduction with Evi1-WT, Evi1-S436A or vector-only, and untransduced cells using the RNA/DNA Purification Micro Kit (Norgen Biotek Corp, Thorold, Ontario, Canada). Libraries were prepared with the Lexogen QuantSeq 3’ mRNA-Seq Library Prep Kit for Illumina (FWD) using an input of 200 ng and performing 14 cycles of amplification. Indexed libraries were then quantified using the Kapa Illumina Library quantification kit (Cat 07960336001) and pooled. 1 × 75 bp sequence reads were generated by clustering 2.0 pM of the library pool on a NextSeq500 High throughput run. Ordered BAM files were generated against the mouse genome feature file Homo_sapiens.GRCh38.90.gtf downloaded from Ensembl (ftp://ftp.ensembl.org/pub/current_gtf). Data analysis was performed using Qlucore Omics Explorer 3.3 (Qlucore, Lund, Sweden) with a FPKM (Fragments Per Kilobase Million) cut-off of 10. We used Deseq2, which converts read counts to a normalised value based using size factors to normalise for differences in the depth of sequence between samples (geometric size factor normalisation method) to calculate fold changes. To capture similarities of changes mediated by Evi1-WT and Evi1-S436A transduction, data analysis was carried out by applying a false discovery rate (FDR)-modified (*p* < 0.05) pair comparison of Evi1-WT vs vector control and Evi1-S436A vs vector control. We applied a group ANOVA for differences in median expression between Evi1-WT and Evi1 S436A and vector-only transduced cells with a *p*-value range of 2.3 × 10^−8^ to 0.01. In order to determine the connectivity of differentially regulated genes within the whole transcriptome of Evi1-WT and Evi1-S436A mutant cells, hypernetworks were used, which allow compression of high dimensional relationships. Manhattan distance matrices were generated for Evi1-WT and Evi1-S436A transduced cells between all transcripts in each transcriptome. Manhattan distances were preferred over Euclidean distances as the former performs better in high dimensions^21^. Selecting only the genes identified as exclusively and significantly regulated in each group, a matrix, *M*, was generated, which described the relationship between differentially expressed genes (*n*^WT^ = 78, *n*^S436A^ = 106) and all other genes (*n*^Total^ = 23766). This matrix was binarised using a threshold of the 30th centile, so that only the closest relationships (smallest Manhattan distances) were retained. Multiplication of matrix *M* by the transpose of this matrix *M*^*T*^ results in a square hypernetwork matrix *M* × *M*^*T*^ whose values represent the number of binary relationships shared between a pair of genes. Connectivity was defined as the mean value for each hypernetwork. To assess organisation of the connections in these networks, entropy was calculated per gene in each hypernetwork. In order to test whether connectivity of each set of genes was greater than expected by random chance, a randomized iterative approach was used. Hypernetworks were generated 1000 times in each cell type, using a randomly selected set of genes (*n*^WT^ = 78, *n*^S436A^ = 106) each time, and connectivity and entropy were calculated.

### Mass spectrometry (MS)

For MS analysis, EVI1 was immunoprecipitated from 6 × 10^8^ SB1690CB AML cells. Following gel electrophoresis, EVI1 containing bands were excised and trypsin-digested. Peptides were separated by liquid chromatography prior to electrospray mass spectrometry on a 4000 Q-TRAP mass spectrometer (AB Sciex, Warrington, UK). MS/MS data were interrogated using MASCOT and confirmed by manual inspection of spectra. Interactome analysis was carried out on in-gel digested samples of EVI1-immunoprecipitate Flag-IP of Flag-tagged EVI1-WT or EVI1-S436A transfected HEK293 cells using standard methodologies (Supplementary Material and Methods).

### In silico analysis of kinase prediction and protein modelling

Kinase prediction: Kinase prediction for EVI1 S436 phosphorylation was carried out using the PHOSHONET platform^22^. Protein modelling: To model the structure of the 426-598aa region of EVI1 the Iterative Threading ASSEmbly Refinement (I-TASSER) was used (https://zhanglab.ccmb.med.umich.edu/I-TASSER/)^23^. Five structures were predicted for EVI1 and assessed by C- and TM-score (confidence scores for estimating the quality of predicted models by I-TASSER). The model prediction with maximum C-score (−0.94) and TM-scores (0.60 ± 0.14) was selected, quantifying the accuracy of the model built. The EVI1-CtBP1 interaction was simulated through modelling of protein-protein docking using ClusProserver applying the initial coordinates of the structure of CtBP1, (28–378), which has largely been resolved (PDB:6CDR)^24,25^. Proteins were in silico positioned in a cubic periodic box with each side at least 1 nm away from the protein. The complexes were parameterized using GROMOS 54A7 force field in a cubic box solvated with SPC water model^26,27^. Both CtBP-binding motifs were used when setting attraction in the docking parameters in ClusPro. In order to examine the phosphorylation at S436 of EVI1, the ViennaPTM tool was used to modify EVI1-CtBP1 PDB file and obtain force-field parameters for phosphorylated EVI1-CtBP1^28^. A neutral charge was introduced at 150 mM NaCl. Long-range interactions were defined using the particle mesh Ewald (PME) algorithm^29^. Energy minimization was carried out using steepest descent after applying position restraints to heavy atoms. This was followed by a 100 ps NVT ensemble at 300 K, and a100ps NPT ensemble at 300 K and 1 bar^30,31^. Production MD was performed at 300 K and 1 bar for 400 ns with frames written every 2 pico seconds. GROMACS modules, such as gmx *rms*, gmx *rmsf* and gmx energy, were used to analyse the stability and behaviour of each system^32–34^. Root Mean Square Fluctuation (RMSF) was calculated for of Cα atoms coordinates of EVI1 in the EVI1-CtBP1 complex, and the p(S436) EVI1-CtBP1 complex in the last 350 ns to ensure the complex reached equilibrium. The g_mmpbsa tool was used to calculate the binding free energy of EVI1-CtBP1 complex and the contribution of S436 to the binding energy by means of energy decomposition^33^. All molecular dynamics (MD) work was generated through HighPerformance Computing facility (Barklacluster, University of Liverpool) using Gromacsv.5.1.4^29,32^.

## Results

### Dynamic EVI1 phosphorylation at serine 436 (S436)

We analysed by mass spectrometry (MS) immunoprecipitated EVI1 from SB1690CB AML cells, which express high levels of EVI1 and ΔEVI1^35^ (Fig. 1a). We identified the EVI1 peptide DKVSPLQNLASINNK (aa 433–447) (NCBI accession: NP_001098548.2), unmodified (Fig. 1b), and in a phosphorylated form at serine S436 (Fig. 1c), in addition to the previously reported EVI1 phosphorylation sites S196, S858 and S860^15,16^. This confirmed EVI1-S436 phosphorylation listed in other studies of cell lines and clinical samples^13,36^. The presence of EVI1-peptides, both phosphorylated, and unmodified at S436, implies a dynamic process involving the region of EVI1 situated adjacent to a putative nuclear localization site (NLS, KEKFKENGKMFKDK aa 421–434), and close to the two CtBP-binding domains of EVI1 (PFDLT aa 553–559, and PLDLS aa 584–590) (Fig. 1a).

### S436 available for phosphorylation is required for EVI1-mediated haematopoietic self-renewal

**Fig. 2:**
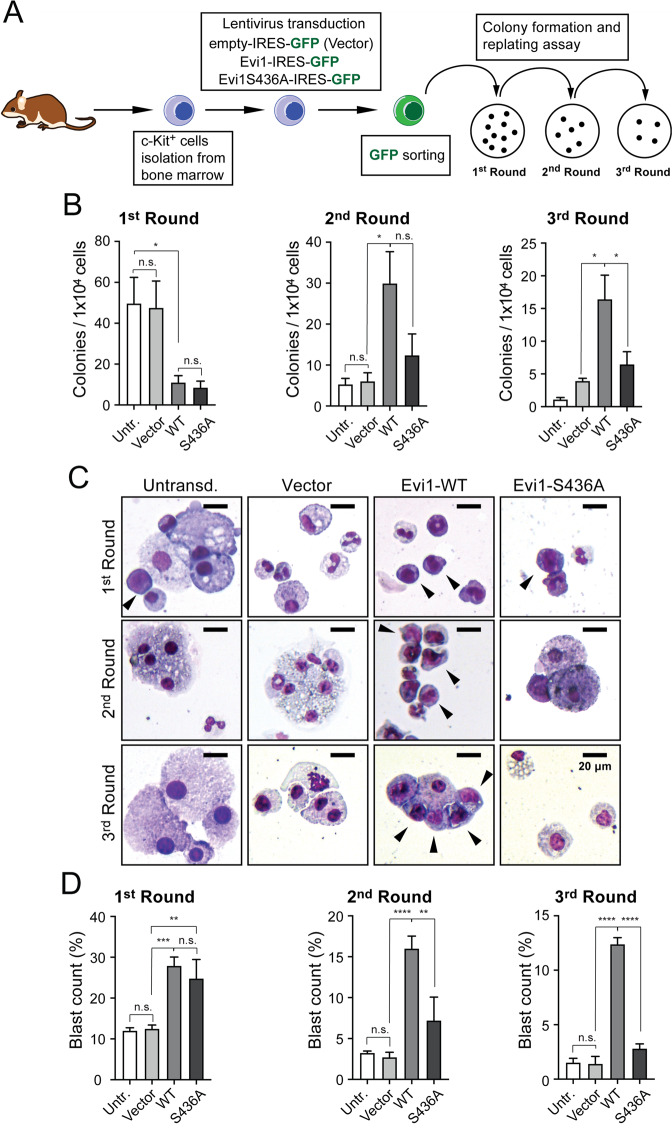
EVI1 with S436 available for phosphorylation is required for EVI1-mediated haematopoietic self-renewal. **a** Schematic representation of the replating assay of haematopoietic progenitors. Immunomagnetic sorted murine haematopoietic c-Kit^+^ stem and progenitor cells (KIT^+^ HSPCs) cells were lentivirally transduced, GFP- FACS selected and plated. After 7 days, colonies were counted and cells harvested for replating (2nd round). The procedure was repeated for a 3rd round. **b** Colony counting (*n* = 6, 6 different mice) after 1st round (left), 2nd round (middle) and 3rd round (right). Statistical analysis: ordinary one-way ANOVA with Tukey post-test. Mean ± SD, **p < 0.01, ***p < 0.001, ****p < 0.0001, n.s. = non-significant). **c** May–Grünwald Giemsa stain of cells from colonies after first, second and third rounds. Black arrowheads point at cells with typical blast morphology. **d** Blast counts from the 1st, 2nd and 3rd round of replating. Statistical analysis: one-way ANOVA, Tukey posttest (mean ± SD, *p < 0.05; ***p < 0.001).

To study the role of phospho-S436 EVI1 we first generated a non-phosphorylatable version of EVI1: EVI1-S436A (Supplementary Fig. 1). To confirm minimal effect of the EVI1-S436A mutation predicted in silico by *PredictProtein*^37^, we compared its localization, *PLZF* and *FOZ* promotor repression, and Rat-1 fibroblast transformation with EVI1-WT as previously described (Supplementary Methods)^15^. We observed no differences in all the above functional readouts (Supplementary Fig. 1d–j). By contrast, in serial replating assays of murine haematopoietic c-Kit^+^ stem and progenitor cells (KIT^+^ HSPCs), EVI1-WT was significantly more efficient in sustaining proliferation and preventing differentiation of blast cells than EVI1-S436A (Fig. 2a, b) with equivalent levels of retroviral transduction and EVI1 expression (Supplementary Fig. 2). Expression of EVI1-WT led to sustained clonogenic activity of KIT^+^ HSPCs by comparison with untransduced or empty vector transduced cells over three rounds, as previously shown^15,16,18^. However, by comparison with EVI1-WT expressing cells, there were significantly fewer EVI1- S436A expressing colonies beyond round two, suggesting an essential function for EVI1-S436 site available for phosphorylation in sustaining EVI1-mediated self-renewal of murine KIT^+^ HSPCs (Fig. 2a, b). Reflecting this, EVI1-WT expression conferred a higher frequency and persistence of blasts from round two onwards. Conversely, EVI1-S436A transduced cells exhibited substantially higher levels differentiated cells with an increased macrophage count (Fig. 2c, d, and Supplementary Fig. 2f).

### Phosphorylatable S436 EVI1 directs transcriptional changes associated with self-renewal

**Fig. 3:**
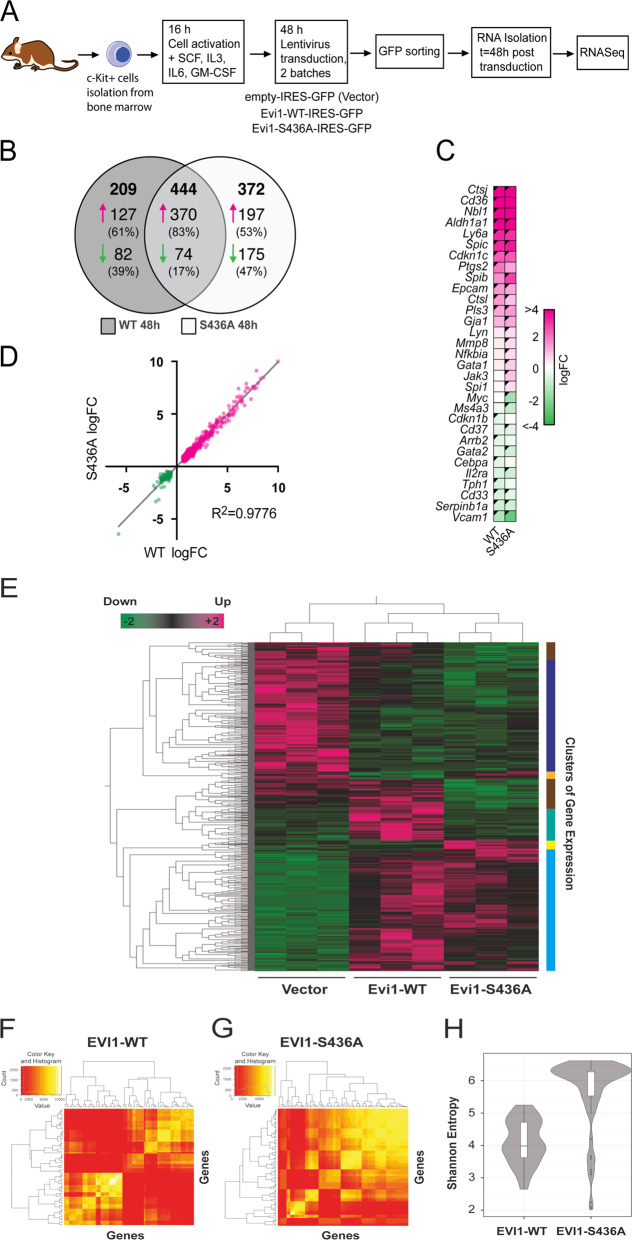
Gene expression is differentially modulated by EVI1-WT and EVI1-S436A. **a** Experimental design. Murine haematopoietic c-Kit^+^ stem and progenitor cells (KIT^+^ HSPCs) cells were isolated and transduced with Evi1-WT and Evi-S436. 48 h after transduction RNA was extracted and processed for RNAseq analysis. **b** Two-way RNAseq analysis of Evi1-WT vs vector-only and Evi1-S436A vs vector-only transduced KIT^+^ HSPCs. Venn diagram of transcripts significantly changed by Evi1-WT or Evi1-S436A (adj. *p* > 0.05, FC > 0.6). **c** Heatmap illustration of selected differentially expressed genes in the two-way comparison (black triangle: adj.*p* < 0.05) **d** Regression analysis comparing fold change of significantly (*p* < 0.05) changing transcripts in Evi1-WT and Evi1-S436A transduced cells. **e** Heatmap illustration of group-ANOVA analysis of vector-only-, Evi1-WT - and Evi1-S436 transduced KIT^+^ HSPCs, adj. *p* value range of 2.3e^−8^ to <0.01. **f**, **g** Hypernetwork heatmap for Evi1-WT (*n* = 78 transcripts, mean connectivity = 2376) and Evi1-S436A (*n* = 106 transcripts, mean connectivity = 3561), respectively. Colour intensity represents number of binary relationships shared between a pair of transcripts with the rest of the transcriptome (*n* = 23766 total transcripts) **h** Shannon entropy in Evi1-WT (*n* = 78) and Evi1-S436A (*n* = 106) induced hypernetworks (*p* < 2.2e^−16^).

To investigate the effect of phosphorylatable S436 on the transcriptome, we expressed EVI1-WT and EVI1-S436A in KIT^+^ HSPCs for 48 h and compared the effects of transcriptional patterns by RNA sequencing (RNAseq) (Fig. 3a). Vector-only transduced and untransduced control cells in biological triplicates were also analysed (Supplementary Excel Table 1). Unsupervised principal component analysis of gene expression patterns showed tight clustering of replicates for EVI1-S436A transfected samples, while in concordance with availability of S436 for phosphorylation EVI1-WT-transduced replicates clustered with a wider distribution and partial overlap with EVI1-S436A (Supplementary Fig. 3a). We first captured common transcriptional changes mediated by EVI1-WT and EVI1-S436A quantitatively and performed a group comparison between EVI1-WT and vector-only transduced cells. We found 653 genes significantly changing, 497 upregulated and 156 downregulated applying a cut-off log-fold change (FC) of >0.6 and a false discovery rate (FDR)-adjusted *p*-value of <0.05 (Fig. 3b, Supplementary Excel Table 1, sheet 2). Amongst these were known EVI1-regulated genes such as the stem cell marker *Aldha1a*, and more than half of the top 40 upregulated genes identified in a previous study investigating the effect on *Evi1*-transduction on murine haematopoietic precursors^18^. In this group *Cepba* was also downregulated, previously shown to be repressed by *Evi1-*mediated interference in myeloid maturation^38^ (Fig. 3c). Comparing EVI1-S436A with vector-only controls, expression of 816 genes changed significantly (567 upregulated, 249 downregulated) (Fig. 3b). Strikingly, 444 of these genes changed concordantly with EVI1-WT, including *Aldh1a1* (Fig. 3b, c, Supplementary Excel Table 1, sheet 2). Among 372 genes changing significantly exclusively with EVI1-S436A we identified upregulation of *Spi1*, which is involved with *Evi1*-driven myeloid haematopoietic skewing^39^, and downregulation of *Ms4a3*, of which repression has previously been implicated in EVI1-mediated malignant progression^40^. Also *Gbp6, Nqo1* and *Cdh17*, which were previously shown to be regulated by EVI1 in murine progenitor cells^18^, were significantly changed exclusively via the non-phosphorylatable Evi1-S436A. We noted that many genes, including *Spi1 and Ms4a3* showed concordant changes both with EVI1-WT and EVI1-S436A, albeit not reaching significance in one or the other group (Fig. 3c), further illustrating a broad overlap of EVI1-WT and EVI1-S436A mediated gene expression patterns after 48 h. In order to delineate expression patterns that significantly discriminate *Evi1-WT* from *Evi1-S436A* transduced cells, we next applied a group ANOVA test to the dataset: 620 genes discriminated EVI1-WT, EVI1-S436A and vector-only transduced cells with a *p* value range of 2.3 × 10^−8^ to <0.01 (Fig. 3e). Significantly and exclusively modulated by EVI1-WT, were 78 genes. Of these, 64 genes were upregulated (Fig. 3e, green cluster, Supplementary Excel Table 1, sheet 3), and 14 downregulated (Fig. 3e, orange cluster). Significantly and exclusively upregulated by EVI1-S436A were 19 genes (Fig. 3e, yellow cluster), and repressed exclusively by Evi1-S436A were 87 genes (Fig. 3e, brown clusters). Comparing Evi1-WT (with EVI1-436 available for phosphorylation) with Evi1-S436A transduction, we can demonstrate that genes exclusively and significantly regulated by EVI1-WT displayed a significantly narrower diversity of connections than EVI1-S436A (*p* < 2.2 × 10^−16^) (Fig. 3f, g). Moreover, a hypernetwork generated with the EVI1-S346A mutant regulated patterns had significantly higher entropy than EVI1-WT regulated (*p* < 2.2 × 10^−16^), demonstrating that coordination of the higher connectivity between the genes in S436A regulated patterns is low (Fig. 3h). It follows that the effect on the entire transcriptome conferred by *Evi1-WT* transduction, and mediating self-renewal, has a significantly more coordinated and focussed effect than that of non-phosphorylatable EVI1-S436. Hypernetwork connectivity and entropy were greater with Evi1-S436A transduction than in hypernetworks of the same size generated from randomly selected genes iterated 1000 times (*p* < 2.2 × 10^−16^) (Supplementary Fig. 3b, c).

### EVI1-S436 phosphorylation negatively affects interaction with CtBP1

**Fig. 4:**
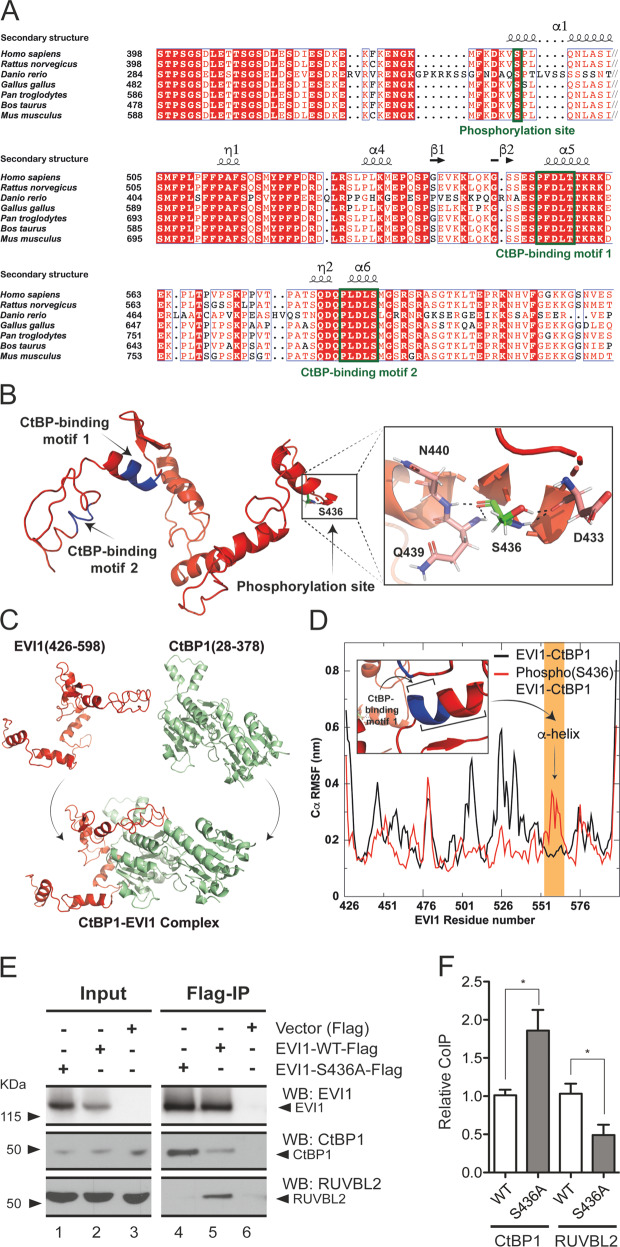
Modelling of EVI1-S436 phosphorylation on CtBP1 affinity. **a** Alignment for the full-length amino acid sequences of EVI1 in Homo sapiens (Uniport Q03112), Mus musculus (Uniport P14404), Rattus norvegicus (Uniport D3ZM26), Danio rerio (Uni- port F1Q834), Gallus gallus (Uniport A0A3Q2U4Z4), Pan troglodytes (Uniport A0A2I3RS65) and Bos taurus (Uniport A0A3Q1LI16) were aligned using MUSCLE and visualized by ESPript and aligned. **b** Three-dimensional prediction of the I-TASSER generated structure of the EVI1 region as in **a**. Both CtBP-binding motifs (blue) and the S436 site are part of predicted *α-*helix structures. Hydrogen bonds formed by Ser436 with Gln440, Gln439 and Asp433 residues (dashed lines). **c** I-TASSER-generated model of EVI1(aa426-598) for modelling the interaction between EVI1 and CtBP1 using ClusPro server. Both CtBP-binding motifs were used when setting attraction in the docking parameters in ClusPro. **d** Modification of the complex according to the force-field parameters of GROMOS 54A7 for effect of EVI1-S436 phosphorylation. Molecular dynamics simulations (400 ns) for modelling EVI1 complexation with CtBP1. Root mean square fluctuation (RMSF) plot of Cα atoms of EVI1 in EVI1-CtBP1 complex and phospho(S436) EVI1-CtBP1 complex. Yellow: region of EVI1 structure (553-566) affecting the *α*-helix stability containing the CtBP1-binding motif 1 (553-557). **e** Experimental confirmation: HEK293 cells transfected with flag-tagged Evi1-WT or Evi1-S436A; protein extracts were subjected to Flag-magnetic beads immunoprecipitation to quantify EVI1-CtBP1 co-Immunoprecipitation. Quantitation of immunoprecipitated RUVBL2 used as a control. **f** Quantitation of independent Co-IP assays (mean ± SD, *n* = 3, unpaired, two-tailed Student’s *t*-test *p < 0.05) as shown in **e**.

Given the location of S436 between the EVI1 zinc finger motifs, and equal promotor affinity with respect to *PLZF* and *FOS* repression with WT and S436A mutated EVI1, we hypothesized that the S436 phosphorylation directs gene expression patterns not by differential DNA binding, but by interaction with other transcriptionally active proteins. A role of S436 phosphorylation for protein interactions was also considered, because S436 is in close proximity to the EVI1-binding sites for the co-repressor CtBP1, which has been shown to be essential for EVI1-mediated haematopoietic self-renewal^41–43^ (Fig. 1). We hypothesized that the S436 phosphorylation might mediate CtBP1 binding, as only EVI1-WT with S436 available for phosphorylation conferred self-renewal. While the tertiary structure of EVI1 is not fully resolved and there is little homology of EVI1 with other proteins, the interaction of EVI1 with CtBP1 has been studied in some detail^41–43^. We therefore modelled the 172aa 426-598 region of EVI1. Cross species sequence alignment of the sequence of the entire region reveals strong conservation of S436 and both CtBP1-binding motifs, implying an essential role for structure and function of EVI1 (Fig. 4a). We next used Iterative-Threading Assembly Refinement (I-TASSER) to model the structure with the highest confidence score^23^. This predicted several α-helix formations involving S436 and both CtBP1-binding sites (Fig. 4a, b). As the crystal structure of CtBP1 has been largely resolved^24,44,45^, we modelled the docking of the predicted 172aa 426-598 structure of EVI1 with CtBP1 using ClusPro (Fig. 4c)^25^. We simulated the effect of S436 phosphorylation on the dynamics of the CtBP1-EVI1 interaction using *Gromacs* with 400 ns molecular dynamics (MD)^29,33,34^. This predicted a stable α-helix configuration within the CtBP1-binding site 553-557 (Fig. 4a) for the CtBP1 docking formation modelled with residues 28–378 (Fig. 4a, b). However, EVI1 S436 phosphorylation is predicted to destabilise this α-helix formation, which may potentially affect the interaction between EVI1 and CtBP1 (Fig. 4d). We calculated the effect of the binding affinity of the 172aa 426–589 region of EVI1 using Molecular Mechanics-Poisson Bolzmann Surface Area (MM-PBSA)^33^. Estimation of the binding energy for the EVI1-CtBP1 complex shows that EVI1- S436 phosphorylation unfavourably shifts the binding energy by approximately 100 kJ/mol (ΔG_binding_ > 0) (Supplementary Fig. 4a), mainly caused by the phosphate group affecting the electrostatic energy. Unphosphorylated EVI1 at S436 in the EVI1-CtBP1 complex contributes favourably (ΔG_binding_ < 0) to the binding energy (Supplementary Fig. 4b), but after phosphorylation the mean contribution of S436 shifted unfavourably from −2.9 KJ/mol to 19.47 KJ/mol. In summary, contrary to our hypothesis, dynamic protein interaction modelling predicted a negative effect of S436 phosphorylation on the affinity of EVI1 for CtBP1. To experimentally verify this prediction, we quantitatively co-immunoprecipitated CtBP1 with Flag-tagged EVI1-WT and EVI1-S436A. We found a significantly increased association of non-phosphorylatable EVI1-S436A with CtBP1, in line with the in silico prediction (Fig. 4e, f). To exclude the possibility that higher CtBP1 affinity of EVI1-S436A was nonspecific, we also quantified the co-immunoprecipitated AAA-ATPase RUVBL2 protein, which also interacts with EVI1^13^, and is functionally relevant for other leukaemogenic transcription factors^46,47^. We showed significantly higher affinity of phosphorylatable EVI1-WT to RUVBL2 compared with non-phosphorylatable EVI1-S436A (Fig. 4e, f).

### Preferential association of EVI1-WT with target-specific kinases and DNMT3A

**Fig. 5:**
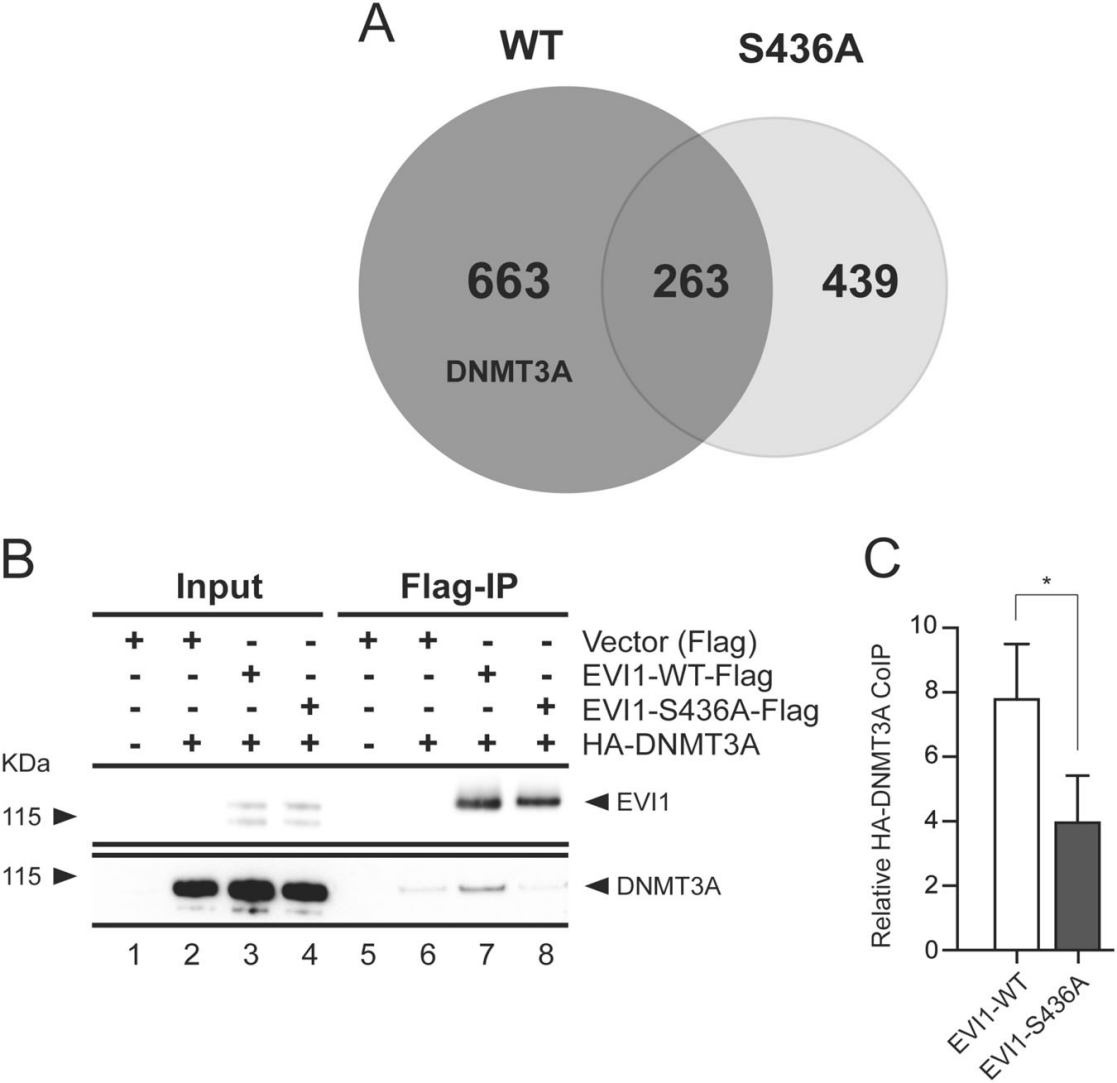
Protein association of EVI1-WT and EVI1-S436A. **a** Venn diagram showing the number of proteins associated with EVI1- WT (dark grey) or EVI1-S436A analyzed by affinity purification and mass spectrometry of co-immunoprecipitated proteins. **b** Co-immunoprecipitation of Flag-tagged EVI1-WT and EVI1-S436A, and HA-tagged DNMT3A in HEK293 cells and western blotting. **c** Signal quantitation of co-immunoprecipitated DNMT3A (mean ± SD, *n* = 3, unpaired, two-tailed Student’s *t*-test; *p < 0.05).

To investigate more broadly the effect of phosphorylatable S436 on EVI1-protein affinity and to determine possible kinases involved, we expressed FLAG-tagged EVI1-WT and EVI1-S436A in HEK293 cells and carried out affinity purification by FLAG-IP and MS analysis versus empty vector control. We considered another MS study of MECOM-encoded proteins, in addition to previously described protein interactions for our data analysis^6,13^ (Supplementary Excel Table 2). In addition, we carried out further analysis using the “CRAPome” platform to consider nonspecific interactions^48^ (Supplementary Excel Table 2, sheet 2). With EVI1-WT and EVI1-S436A we co-immunoprecipitated 926 and 702 proteins, respectively. Of these, 263 proteins co-immunoprecipated with both EVI1-WT and EVI1-S436A (Fig. 5a). The number of detected proteins is similar to that in the interactome study concerning MECOM-encoded proteins in T47D breast cancer cells^6^, with which there is a considerable overlap: 209 proteins in our dataset (22%) were also detected in the Ivanochko study (Supplementary Excel Table 2, sheet 3). Of these 209 proteins, 89 were detected exclusively in the EVI1-WT interactome, 65 in both IPs, and 55 exclusively in the EVI1-S436A interactome. Of the previously described 102 proteins that interact with EVI1^13^ (Supplemental Excel Table 2, sheet 4), we identified 21 in our dataset, of which 17 were detected exclusively in the EVI-WT IP, and three in both. Only one previously described EVI1-interacting protein (PRDX1) was detected exclusively with EVI1-S436A^13^. Our data demonstrate that phosphorylatable EVI1-WT interacts with more proteins and co-immunoprecipitates with more known EVI1-interactors than EVI1-S436A, supporting the concept that EVI1-S436 phosphorylation serves to facilitate protein recruitment for functional interactions.

Several kinases were identified in the EVI1-WT interactome. An in silico analysis of the DKVSPLQNLASINNK sequence of EVI1, using *PhosphoNe*t^22^ showed that EVI1-S436 is set in a protein sequence that is a putative target of multiple kinases (Supplementary Fig. 5), including CDK2 and CDK3. Both CDK2 and CDK3 were detected in the EVI1-WT interactome. We also detected the casein kinase CSNK2A1, which has previously been implicated in EVI1 phosphorylation^13^. Only a few of the proteins that co-immunoprecipitated with EVI1 have been investigated functionally for potential biological relevance, and for most, a role in haematopoietic self-renewal is elusive. However, in the EVI1-WT, but not in the EVI1-S436A interactome, we detected the DNA methyltransferase DNMT3A. DNMT3A has an essential role in haematopoiesis, interacts with EVI1^49,50^, and has been suggested to mediate EVI1-directed methylation patterns in AML^20^. To confirm this MS finding, we transfected HEK293 cells with HA-tagged DNMT3A together with EVI1-WT or EVI1-S436A. Quantitative co-immunoprecipitation of EVI1 and DNMT3A demonstrated a higher affinity of EVI1-WT with DNMT3A compared with EVI1-S436A (Fig. 5b, c), corroborating the MS findings.

### S436 available for phosphorylation is required for EVI1-mediated DNA-methylation patterns

**Fig. 6:**
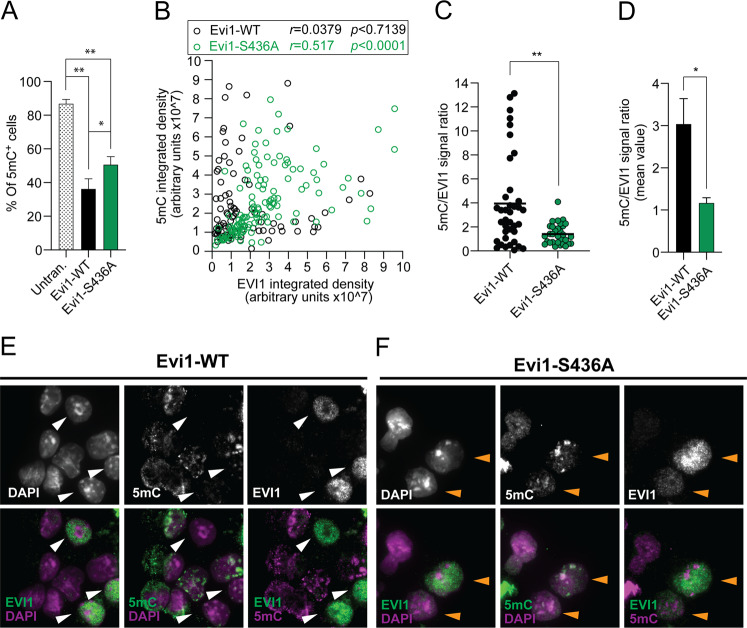
S436 available for phosphorylation is required for EVI1-mediated DNA-methylation patterns. **a** Quantitation of total 5-mC^+i^ in KIT^+^ HSPCs cells after transduction with Evi1-WT or Evi1-S436A. Cells were scored by presence of absence of 5-mC IF stain (one-way ANOVA, Tukey post-test, mean ± SD, *p < 0.05, **p < 0.01). **b** Correlation of the EVI1 and 5-mC signals by Pearson’s test (r range −1 for total exclusion, 1 for total association) for EVI1-WT (black circles) and EVI1-S436A (green circles). **c** Signal ratio of Evi1 and 5-mC (*t*-test; **p < 0.01). **d** Quantitation as in E of 3 independent experiments (mean ± SD, *p < 0.05). **e**, **f** Top panels illustrate individual stains (gray scale) of EVI1, DAPI and 5-mC, bottom panels merged. Arrows indicate cells in corresponding upper and lower panels with high EVI1-WT signal and absent 5 mC signal (white), and cells with high EVI1-S436A signal cells with high EVI1 signal and high 5 mC signal (orange).

The methyltransferase DNMT3A is an important mediator of de novo cytosine DNA methylation in haematopoietic self-renewal and differentiation. Loss of DNMT3A function in haematopoietic progenitor cells impairs differentiation and promotes self-renewal^49^. Acquired mutations in DNMT3A, which prevent the formation of catalytically active DNMT3A tetramers^51^, are common in AML. In non-leukaemic haematopoietic progenitors with inherited *DNMT3A* disruption focal hypomethylation is a distinctive feature, while CpG-island hypermethylation in AML is a consequence of leukaemic progression^52^. We demonstrated distinct 5-mC staining in untransduced KIT^+^ HSPCs in most cells with speckled signal as previously described^53^ (Supplementary Fig. 6). As DNMT3A preferentially interacts with EVI1-WT compared with EVI1-S436A, we investigated if *Evi1-W*T or *Evi1-S436A* transduction affects the 5-mC staining patterns. With *Evi1-WT* transduction, but not with *Evi1-S436A*, we noted a distinct population of entirely 5-mC-negative cells (Fig. 6a). To quantify this observation, we correlated the signal distribution of 5-mC- with the EVI1 signal in Evi1-WT and Evi1-S436A transduced KIT^+^ HSPCs. In Evi1-WT transduced cells the signal was not correlated with the 5-mC staining (*r* = 0.0379) (Fig. 6b, e), caused by the distinct cell population with high EVI1 signal, but low or absent 5-mC (Fig. 6c, d). The absence of this cell population in *Evi1-S436A* transduced cells resulted in a significantly higher correlation of the 5-mC with the EVI1-S436A signal (Fig. 6b, f) (*r* = 0.517, range −1 for total exclusion, 1 for total association). Together, these results show that self-renewal mediated by EVI1-WT is associated with maintenance of a cell population with low 5-mC staining, which is absent in EVI1-S436A.

## Discussion

EVI1 and other *MECOM*-encoded transcriptional regulators are essential for early development and haematopoiesis, while aberrantly high expression of *EVI1* has potent oncogenic properties. Defining the role of EVI1 in haematopoietic stem cell maintenance will also be important for targeted therapeutic approaches for leukaemia driven by high *EVI1*. There is accumulating evidence for post-translational modifications governing EVI1 function, and phosphorylation is potentially therapeutically targetable. Our MS analysis of endogenously expressed EVI1 in SB1690CB AML cells confirmed S436 phosphorylation, which was previously listed in other EVI1-overexpressing malignancies^13,36^. Presence of both phosphorylated and non-phosphorylated S436 in leukaemia cells indicates a dynamic process, which involves a region of the EVI1 protein that is spliced out in in the ΔEVI1 isoform. DNA binding sites of ΔEVI1 are largely overlapping with EVI1^54^, but ΔEVI1 does not transform, or support normal development^8,9^. We show that the non-phosphorylatable EVI1-S436 mutation, whilst maintaining EVI1-functional readouts with respect to promotor affinity, nuclear localisation and Rat-1 transformation, confers abrogated self-renewal capacity. These data imply that the integrity of this region with the S436 available for phosphorylation is essential for EVI1-mediated haematopoietic self-renewal. Detailed analysis of EVI1-WT and EVI1-S436A mediated expression patterns in KIT^+^ HSPCs confirmed multiple EVI1 target genes^18^, despite employment of a different experimental setup, longer Evi1-exposure after transduction (48 vs 24 h) and another type of RNA analysis. Our analysis, however, further delineates patterns of EVI1-modulated transcriptional profiles, which are partly regulated independently of S436 phosphorylation. Expression patterns mediated by EVI1-WT include genes with an essential role in stem cell maintenance, and show that EVI1 with S436 available for phosphorylation has a much more coordinated effect on the entire transcriptome, implying that the S436 phosphorylation focuses transcriptional patterns towards self-renewal. Recent data from our group and others suggest that many EVI1 functions are regulated by dynamic interactions with other proteins^6,13,15^. To explain the differences in gene expression patterns mediated by EVI1-WT compared with EVI1-S436 we provide evidence for modulation of EVI1-protein interactions by S436 phosphorylation, with increased affinity of non-phosphorylatable EVI1-S436A to CtBP1. With respect to the CtBP1 interaction our results were partly generated using high-end computational modelling approaches, for which we took advantage of the known CtBP1 structure^24,44,45^, and available data with respect to EVI1 and CtBP1 binding^41^. Similar approaches for the detailed analysis of the interaction of EVI1 with other proteins (e.g. RUVBL2 and DNMT3A, see below) would be illuminating, but lacking exact information about these proteins’ EVI1-binding sites, this is currently not possible in the same way. The finding that EVI1-S436 phosphorylation negatively affects affinity to CtBP1 was counterintuitive, as CtBP1 interaction is essential for EVI1 function, and only EVI1-WT with S436 available for phosphorylation confers self-renewal. These data, together with our previous observation concerning a modulation of CtBP1-association also via the carboxy-terminal SQS-phosphorylation^15^, supports the concept of a dynamic and kinase-governed complex and finely regulated EVI1 interaction with CtBP1, and applies also to other interactions, of which detailed understanding might be therapeutically important for EVI1-overexpressing malignancies. We investigated the RUVBL2 interaction as a control for CtBP1, since this AAA-ATPase has been shown to interact with EVI1 previously^13^, and is of functional importance for other leukaemogenic transcriptional regulators^46,47^. Preferential association of EVI1-WT with RUVBL2 warrants further investigations into the role of RUVBL2 for EVI1-mediated self-renewal. Our MS-protein interaction studies implicate CDK2 and CDK3 as likely kinases for S436 phosphorylation, as they co-immunoprecipitated with EVI1-WT and share the S436 target sequence. Selective and quantitative detection of S436-phosphorylated and non-phosphorylated EVI1, for example by a specific antibody and treatment with selective CDK3 and other inhibitors would be necessary for further investigations, particularly since as CDK3 has a role for haematopoietic self-renewal in response to chemotherapy^55^. Having demonstrated preferential interaction of EVI1-WT with DNMT3A, we show that EVI1-WT affects DNA-methylation patterns in haematopoietic progenitor cells as assessed by 5-mC staining. Our data with respect to the maintenance of a population with low or absent 5-mC only by Evi1-WT suggests an interference of EVI1 with de novo methylation. We are currently further characterising the hypomethylated cell population maintained by *Evi1-WT* transduction, and the genes affected by differential methylation patterns, also in relation to differentially expressed transcripts in the RNAseq analysis. We further investigate how the interaction with aberrantly high levels of EVI1 affects tetramer assembly and function of DNMT3A in normal haematopoietic progenitor and transformed leukaemic cells, which also might be relevant in the context of therapy with hypomethylating agents. Moreover, since the other MECOM-encoded protein MDS-EVI1 (PRDM3) also contains the S436 motif, investigations whether and how S436 phosphorylation affects the MDS-EVI1 protein are also required. In summary, our data provide evidence of an important role of EVI1-S436 and its availability for phosphorylation for haematopoietic self-renewal via modulation EVI1-protein interactions associated with transcriptional changes, and alteration of EVI1-directed DNA methylation. Aberrantly high expressed EVI1 appears to dynamically alter the composition and stoichiometry of transcriptional regulatory complexes via its S436 phosphorylation. Targeting the kinases promoting S436 phosphorylation is worthy to investigate further for therapeutic benefit in EVI1-driven leukaemia.

## Supplementary information

Paredes et al Suppl. materials and methods Rev 1

Suppl. Figure 1

Suppl. Figure 2

Supplmentary Figure 3

Supplementary Figure 4

Suppl. Figure 5

Supplementary Figure 6

Supplementary Excel Table 1 - RNA seq

Supplementary Excel table 2 -Interactome
