# Supplementary material for: EVI1 phosphorylation at S436 regulates interactions with CtBP1 and DNMT3A and promotes self-renewal
Source: Cell Death Dis. 2020 Oct 20;11(10):878. doi: 10.1038/s41419-020-03099-0 (PMC7576810; doi:10.1038/s41419-020-03099-0)
Supplement: Supplementary file 1 — Paredes et al Suppl. materials and methods Rev 1 [file 41419_2020_3099_MOESM1_ESM.docx]

Paredes *et al* **EVI1-phosphorylation at S436 is required for haematopoietic self-renewal and modulates interactions with CtBP1 and DNMT3A**

**Supplementary materials and methods**

**Supplementary Table S1: Antibodies (in addition to the antibodies detailed in the main text)**

| **Antigen** | **Company** | **Dilution range used** | **Catalogue No** |
| --- | --- | --- | --- |
| GAPDH | Ambion | 1:20,000 to 1:100,000 |  |
| RUVBL2 | NOVUS | 1:2,000 | #NBP1-40354 |
| Histone H_3_ | Cell Signaling Technology | 1:5,000 | #9715 |
| DNAMT3A (A-10) | Santa Cruz Biotechnology | 1:200 to 1:1,000 | #SC-373905 |
| 5-mC | Bio-Rad | 1:200 to 1:1,000 | #MCA2201 |
| CtBP1 | BD Biosciences | 1:2,000 | #612042 |
| EVI1 | (C50E12) Cell Signaling Technology | 1:1,000 to 1:5,000 | #2593 |
| FLAG | Anti-FLAG^®^ M2 Magnetic Beads Sigma | 50 μL beads suspension /mL protein extract | # M882 |
| Mouse IgG | Anti-mouse IgG HRP Linked Whole Ab, Amersham ECL, GE Healthcare | 1:5,000 to 1:10,000 | #NA931V |
| Rabbit IgG | Anti-rabbit IgG HRP Linked Whole Ab, Amersham ECL, GE Healthcare | 1:5,000 to 1:10,000 | #NA9340V |
| Mouse IgG | Goat anti-mouse IgG (H+L) Secondary antibody, Alexa Fluor 594 conjugated, Life Technologies | 1:800 to 1:2,000 | #A-11032 |
| Rabbit IgG | Goat anti-rabbit IgG (H+L) Secondary antibody, Alexa Fluor 488 conjugated, Life Technologies | 1:800 to 1:2,000 | #A-11034 |

***Supplemental Methods***

**Western blot quantification**

Chemo luminescent signals of western blot images were acquired utilising the ChemiDoc System (BioRad). Digital images were generated form acquisitions of sequential exposures in 5 to 30-second intervals for up 30 minutes (depending on signal intensity) and exported as JPEG or TIFF files. Image analysis was carried out using the ImageJ software. Images were processed in High-Low look up table (HiLo LUT) to visualise and avoid saturated signals. To quantify the signal of specific bands, integrated density was measured over regions of interest (ROI) over each imprinted band using the Analyze tool of ImageJ as described previously^1^. For immunoprecipitation assays quantifications, co-immunoprecipitated proteins levels were normalized against the level of the IP level in each given condition.

**Site-directed mutagenesis**

Site-directed mutagenesis was carried out as described previously^1^ with the primer sequences as in Suppl. Table 2

***Suppl. Table S2: Primer Sequences for site directed mutagenesis***

| **Human EVI1**  **SPLQ to APLQ** |  |
| --- | --- |
| S436A_FW | 5'-GGTAAAATGTTCAAAGACAAAGTAGCCCCTCTTCAGAATCTGGC-3' |
| S436A_RV | 5'-GCCAGATTCTGAAGAGGGGCTACTTTGTCTTTGAACATTTTACC-3' |
| **Mouse codon- optimised EVI1 SPLQ to APLQ** |  |
| APLQ_FW | 5'-TGTTCAAGGACAAAGTCGCCCCCCTGCAGAACCTG-3' |
| APLQ_RV | 5'-CAGGTTCTGCAGGGGGGCGACTTTGTCCTTGAACA-3' |

Global effect of point mutations in the EVI1 protein were predicted with The PredictProtein server^2^ were a machine learning based approach accounts for evolutionary information, structure features and available annotation (whichever is possible) to score the impact of a single residue replacement one by one. The scale goes from -100 (green) for neutral/no effect to 100 (magenta) for a strong effect (Supplementary Figure 1)

**Reporter gene assays**

Reporter gene assays were carried out in stably *Evi1-WT*, *Evi1-S436A* or vector transduced Rat1 cells. Cells were plated in 6-well plates and co-transfected with PLZF-luciferase or FOS-luciferase and TK-renilla luciferase reporter plasmid. Comparable expression of EVI1 protein was monitored by western blot (Supplementary Figure 1). After 48 hours cells were lysed and firefly and renilla luciferase activities were assayed using the Dual-luciferase reporter assay (Promega, Southampton, UK) measured on a Victor3 plate reader (Perkin Elmer, Seer Green, UK).

***Protein sequences alignment***

EVI1 protein sequences from the NCBI database were aligned using ClustalW2 or Clustal Omega program (https://www.ncbi.nlm.nih.gov/pubmed/30976793), and analyzed and illustrated using the Uniprot knowledgebase^3^, and the Jalview software^4^.

***Rat-1 fibroblast transformation assay***

Lentiviral Rat-1 cell transduction was carried out as described^5^. HEK293FT packaging cells were transfected with mouse codon optimised EVI1 lentiviral vectors pRRL.PPT.SF.EVI1mCo.IRES_EGFP.pre and pRRL.PPT.SF.EVI1mCoS436A.IRES_EGFP.pre using X-tremeGENE™ HP DNA Transfection Reagent, Sigma). Lentiviral packaging vectors pHCMV-G, pMDLg/pRRE and pRSV-Rev were co-transfected. Viral particles were produced in OPTIMEM with Glutamax (Life Technologies, Paisley, UK) in two batches at 24 and 48 hours after transfection, filtered with a 0.45 μm filter and concentrated with a Vivaspin 20 centrifugation concentration column (Sartorius AG, Göttingen, Germany). Rat-1 cells were sequentially infected with two viral batches by spinoculation (60 minutes at 1,250 x g at 32ºC) and cultured until visible GFP expression (epifluorescence). Stable transduced cells lines were selected FACS-sorting selection based in the GFP emission (4 rounds of selection, over 98% of GFP^+^ cells). Equal levels of EVI1 expression were confirmed by western blot **(**Supplementary Figure1B). GFP^+^ cells (1x10^4^) were seeded in methylcellulose medium (MethoCult™ M3231, Stem Cell Technology, Cambridge, UK). After 14 days, cell colonies were quantified and documented using a DMIL inverted microscope fitted with a MC170 HD camera (Leica, Wetzlar, Germany).

**Immunofluorescence (IF) analysis**

Rat-1 fibroblasts were seeded at a density of 0.5x10^6^ in 1ml DMEM 10% FBS media onto methanol sterilised 13 mm cover glasses (VWR) in a 24-well plate (Corning) and incubated overnight at 37^o^C. Murine c-Kit^+^ cells were spun at 200 rpm for 2 min (Cytospin 2, Shandon, Fisher Scientific, Loughborough, UK) onto POLYSINE slides (VWR International). Cells were fixed with methanol-free 2% formaldehyde in 1xPBS (Thermo Scientific), washed in 1X PBS and blocked with 5% goat normal serum (Cell Signaling Technology) and 0.3% Triton-X100 (Sigma) containing 1X PBS. Primary and secondary antibodies were used consecutively following standard procedures in a 0.1% BSA (Cell Signaling Technology) and 0.3% Triton-X100 containing 1X PBS. All washes were done with 1xPBST (0.05% Tween-20). For cells stained with anti-5-methylcytosine antibody, cover glasses/slides were denatured for 10 minutes with 200 µL of 2N hydrochloric acid (HCl) in 1x PBS in a 37°C incubator, then neutralised with 200 µL of 0.1M Tris-HCl (pH 8.3) for 10 min at room temperature. Blocking and antibody incubations were carried out as described above. Slides were kept in the dark and stored at 4^o^C until imaged.

Conventional fluorescent microscopy was performed in an Olympus BX51 microscope using an UPlanSApo 100X oil immersion lens and the Q-capturePro7 imaging system (Retiga 6000 camera, QImaging). Reference Regions of interest (ROI) covering the entire positively stained area of the nuclei (DAPI stain) were used to analysed the signal level in the EVI1 and 5-mC stains (50+ cells per condition). We calculate the Integrated density by using the ImageJ software. To discard signal saturated ROIs, the images were analysed using HiLo intensity LUT. Dispersion plot (XY plot) were created in GraphPad prism and Pearson coefficients were calculated to asses signal correlation (free open source Social Science Statistics).

**Mass spectrometry**

Interactome studies: After separation of IP products via SDS-PAGE gel electrophoresis, each lane was separated into fractions. After alkylation and reduction, gel pieces were digested using trypsin overnight, and analysed with an Ultimate 3000 HPLC (Dionex, Sunnyvale, CA) in-line with a TripleTOF 6600 mass spectrometer (Sciex, Warrington, UK). The trapping column was an Acclaim PepMap 100 C18 cartridge (Thermo, Loughborough, UK) and the analytical column was an Acclaim PepMap 100 C18 NV column (Thermo). Buffer A comprised of 98% water, 2% acetonitrile and 0.1% formic acid, buffer B comprised of 80% acetonitrile, 20% water, 0.1% formic acid. Peptides were eluted over a gradient from 0 to 40% buffer B. Peptides were fragmented in a data-dependent manner. Spectral data was searched using MASCOT (version 2.5.1) against the Uniprot human database (downloaded on: 20/06/2018). Downstream analysis and data processing was performed using R (version 3.4.1).

**References:**

1 Paredes, R. *et al.* EVI1 carboxy-terminal phosphorylation is ATM-mediated and sustains transcriptional modulation and self-renewal via enhanced CtBP1 association. *Nucleic Acids Research* **46**, 7662-7674, doi:10.1093/nar/gky536 (2018).

2 Yachdav, G. *et al.* PredictProtein--an open resource for online prediction of protein structural and functional features. *Nucleic Acids Res* **42**, W337-343, doi:10.1093/nar/gku366 (2014).

3 UniProt, C. UniProt: a worldwide hub of protein knowledge. *Nucleic Acids Res* **47**, D506-D515, doi:10.1093/nar/gky1049 (2019).

4 Waterhouse, A. M., Procter, J. B., Martin, D. M., Clamp, M. & Barton, G. J. Jalview Version 2--a multiple sequence alignment editor and analysis workbench. *Bioinformatics (Oxford, England)* **25**, 1189-1191, doi:10.1093/bioinformatics/btp033 (2009).

5 Cante-Barrett, K. *et al.* Lentiviral gene transfer into human and murine hematopoietic stem cells: size matters. *BMC research notes* **9**, 312, doi:10.1186/s13104-016-2118-z (2016).

**Legends to supplemental figures**

**Suppl. Figure 1 *Assessment of EVI1-S436A mutation.* A.** *In silico* prediction of the effect of point mutations in the EVI1 protein. Top panel: whole EVI1 protein; bottom panel: region containing the S436 SQLP motif (dark square). Position of the S436 denoted by asterisk and the alanine substitution by an arrowhead. **B.** Sanger sequencing, confirming mutation of the SPLQ motif to APLQ. **C.** Western blot analysis of EVI1-WT and EVI1 S-436A expression in transduced Rat-1 fibroblast (stable cell lines). **D.** Localisation of endogenously expressed EVI1 in SB1690CB AML cells (upper panel). EVI1 negative OCI-AML5 used as negative control (lower panel)**. E.** EVI1 localisation in *EVI1-WT* and *EVI1-S436* transfected HEK293 cells **F.** EVI1 localisation in *Evi1-WT* and *Evi1-S436* transduced Rat-1 fibroblasts. **G.** EVI1 localisation in *Evi1-WT* and *Evi1-S346* transduced murine cKit^+^ cells. DAPI for DNA staining. Remaining GFP emission seen at the right-hand side panel at figures C and D due to the IRES-GFP vectors. **H.** Schematic representation of vectors used in the luciferase assays**. I:** RT-PCR -quantitation of EVI1-mediated repression of PLZF (left panel) and FOS (right panel) gene promoters (T-test, ns=not significant). **J.** Quantitation of colony formation in stable transduced Rat1 fibroblast with Vector, Evi1-WT or Evi1-S436A. Controls: untransduced (Untransd.) Rat-1 fibroblasts. Statistical analysis: One-way ANOVA, Tukey posttest (C) (mean ± SD, * *p*<0.05; ** *p*<0.01; ns=not significant).

**Suppl. Figure 2 *Sorting and characterisation of transduced murine haematopoietic c-Kit^+^ stem and progenitor cells (KIT^+^ HSPCs)*  A**. Transduced mouse KIT^+^ HSPCs were analysed by flow cytometry, gated and sorted by GFP emission, showing percentages of transduced cell. **B, C.** Median Fluorescence Intensity (MFI) of vector, Evi1- WT and Evi1-S436A sorted KIT^+^ HSPCs cells **D.** Quantitative RT-PCR Evi1-WT and Evi1-S436A expression in sorted mouse KIT^+^ HSPCs cells from A. **E.** Typical murine KIT^+^ HSPCs colony morphology (direct bright field microscopy) in methylcellulose-based medium after 7 days in culture (grey panels). Single picked c-Kit^+^ colonies were stained with May-Grünwald Giemsa stain (colour panels) showing a mixture of progenitor-like cells, others in active cell division and cells already committed with the myeloid differentiation path in the same colonies. **F:** Macrophage count form colonies of EVI1-WT and EVI1-S436 transduced KIT^+^ HSPCs from a single animal at rounds 1, 2 and 3.

**Suppl. Figure 3 *RNA*seq *analysis* A.** Unsupervised principal component analysis of transcriptomic data. Data is processed and regularised log transformed in DEseq2. The top 500 gene based on variance were used to generate the figure. **B.** Iterated connectivity and Hypernetwork connectivity (number of binary relationships shared between a pair of transcripts with the rest of the transcriptome (n=23766)) of a random set of 106 genes iterated 1000 times. Red line indicates the comparison of mean connectivity of the EVI1-S436A hypernetwork (p<2.2e^-16^). **C**. Entropy analyses Shannon entropy calculated from EVI1-S436A hypernetwork compared with hypernetworks generated from 106 randomly selected genes iterated 1000 times (p<2.2e^-16^).

**Suppl. Figure 4 In silico *effect of S436 Phosphorylation on CtBP1 - EVI1 interaction*  A.** Comparative estimation of binding energy (ΔG _binding_) in EVI1-CtBP1 complex with and without EVI1-S436 phosphorylation, calculated using the Molecular Mechanics-Poisson Bolzmann Surface Area (MM-PBSA). Phosphorylation at S436 in EVI1-CtBP1 complex changes the binding energy by 100 kJ/mol. **B.** Contribution of S436 to the binding free energy (ΔG _binding_) calculated by means of energy decomposition, which showed that the average contribution of S436 to the binding energy in EVI1- CtBP1 complex has shifted unfavorably from -2.9 KJ/mol to 19.47 KJ/mol after phosphorylation.

**Suppl. Figure 5. In silico *analysis of the kinase targeting EVI1 protein-sequence containing S436***  Analysis using PhophoNet (Kinexus) (<http://www.phosphonet.ca/>). Listed are the top 15 candidate kinases that phosphorylate the DKVSPLQNLASINNK sequence of EVI1.

**Suppl. Figure 6 *5-mC immunofluorescence (IF).* A.** Mitotic cells of mouse KIT^+^ HSPCs (upper panels) or Rat1 fibroblasts (bottom panels) were stained with the 5mC antibody. DAPI :chromatin. **B.** Mouse c-Kit^+^ cells were stained with the 5-mC antibody. DAPI: chromatin. **C.** Quantitation of 5-mC and EVI1 signals. Reference Regions of interest (ROI) covering the entire positively stained area of the nuclei (DAPI stain), annotated as mask, were used for analysis of signal level of EVI1 and 5-mC. The integrated density was calculated to generate a dispersion plot. **D.** Schematic representation. Cells with high EVI1 and 5-mC signals score a high Pearson’s coefficient (Mask 3). Cells with high EVI1 signal and low 5-mC signal (Mask 1), score a low Pearson’s coefficient.

**Supplementary Excel Tables:**

**Suppl. Table Excel 1: *RNAseq analysis*.** Sheet 1: RNA*seq* gene expression analysis output as FPKM (Fragments Per Kilobase Million) of mouse KIT^+^ HSPCs untransduced, vector only transduced, or transduced with Evi1- WT and Evi1-S436A. Sheet 2: Two-way comparison of vector transduced versus Evi1-WT or Evi1-S436A transduced KIT^+^ HSPCs. Sheet 3: Group ANOVA analysis of gene expression discriminating between vector only, Evi1-WT or Evi1-S436A. Colour coding corresponding to clusters illustrated on Figure 3.

**Suppl. Table Excel 2: *EVI1 interactome analysis.*** Sheet 1: MASCOT analysis of EVI1 co-imunprecipitatied proteins. Listed are proteins that were detected with a MASCOT-score of 20 (grey shading including lower scoring (MASCOT score 15-20) proteins) in either the EVI1-WT or the EVI1-S436A interactome Sheet 2: Analysis using the CRAPOME platform (<https://reprint-apms.org/?q=reprint-home>) providing data concerning detection in other affinity purification experiments and possible not-specific detection. Sheet 3: comparison of data generated in this study with the interactome analysis of EVI1 in Ivanochko *et al.* 2018. Sheet 4: Comparison of data analysis from this study with previously reported EVI1 interacting protein with relevant references.
