# Supplementary figures and images for: EVI1 phosphorylation at S436 regulates interactions with CtBP1 and DNMT3A and promotes self-renewal
Source: Cell Death Dis. 2020 Oct 20;11(10):878. doi: 10.1038/s41419-020-03099-0 (PMC7576810; doi:10.1038/s41419-020-03099-0)

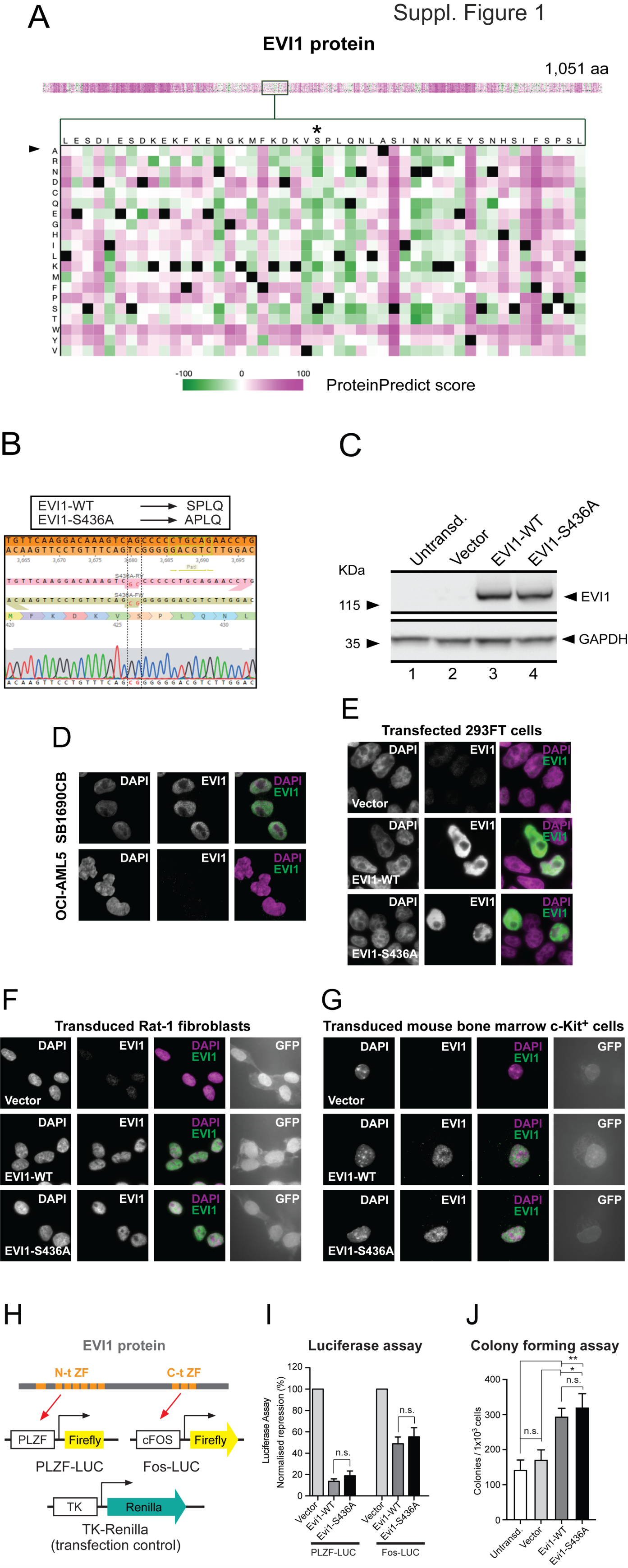

Supplement: Supplementary file 2 — Suppl. Figure 1 [file 41419_2020_3099_MOESM2_ESM.png]

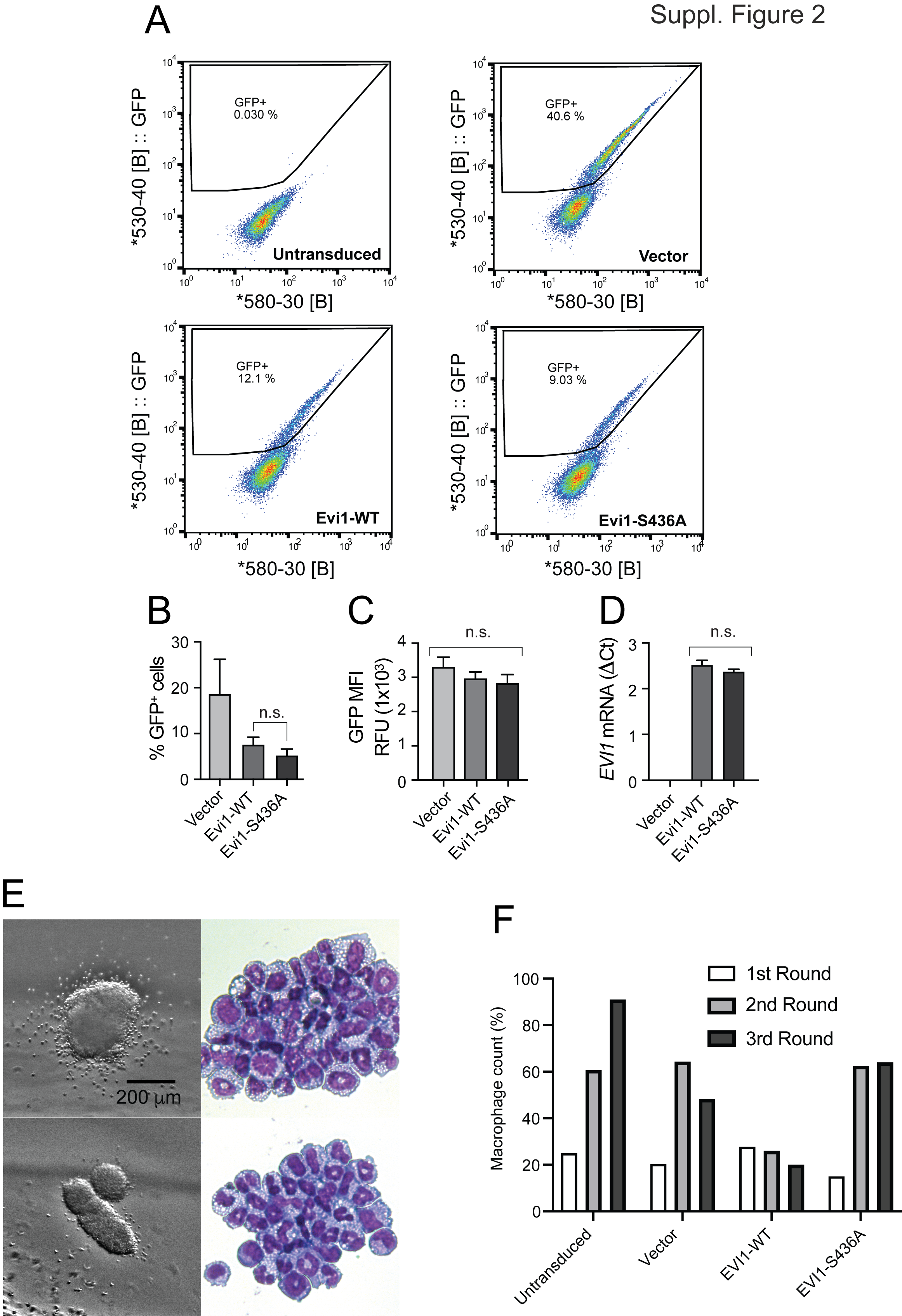

Supplement: Supplementary file 3 — Suppl. Figure 2 [file 41419_2020_3099_MOESM3_ESM.png]

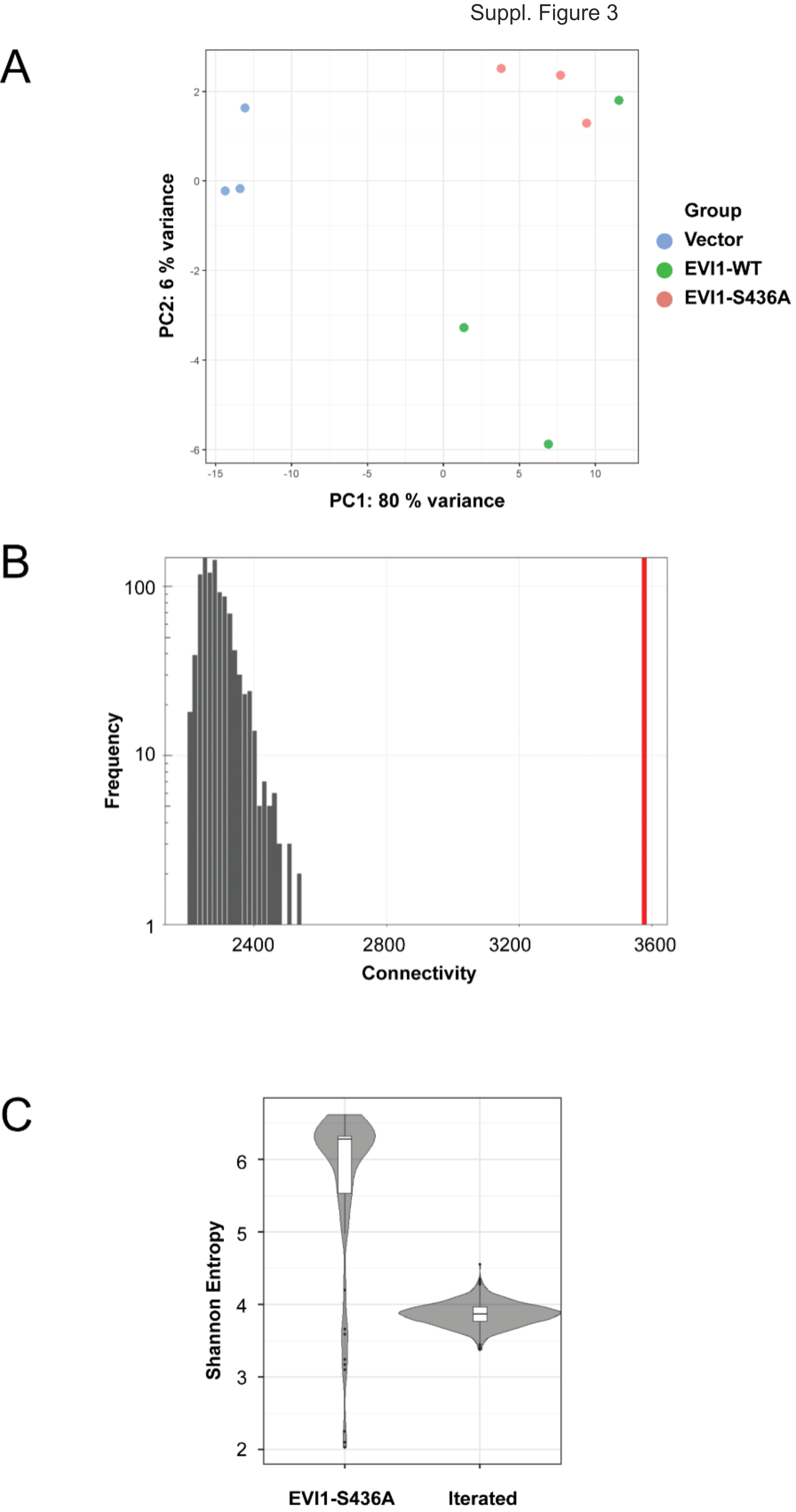

Supplement: Supplementary file 4 — Supplmentary Figure 3 [file 41419_2020_3099_MOESM4_ESM.png]

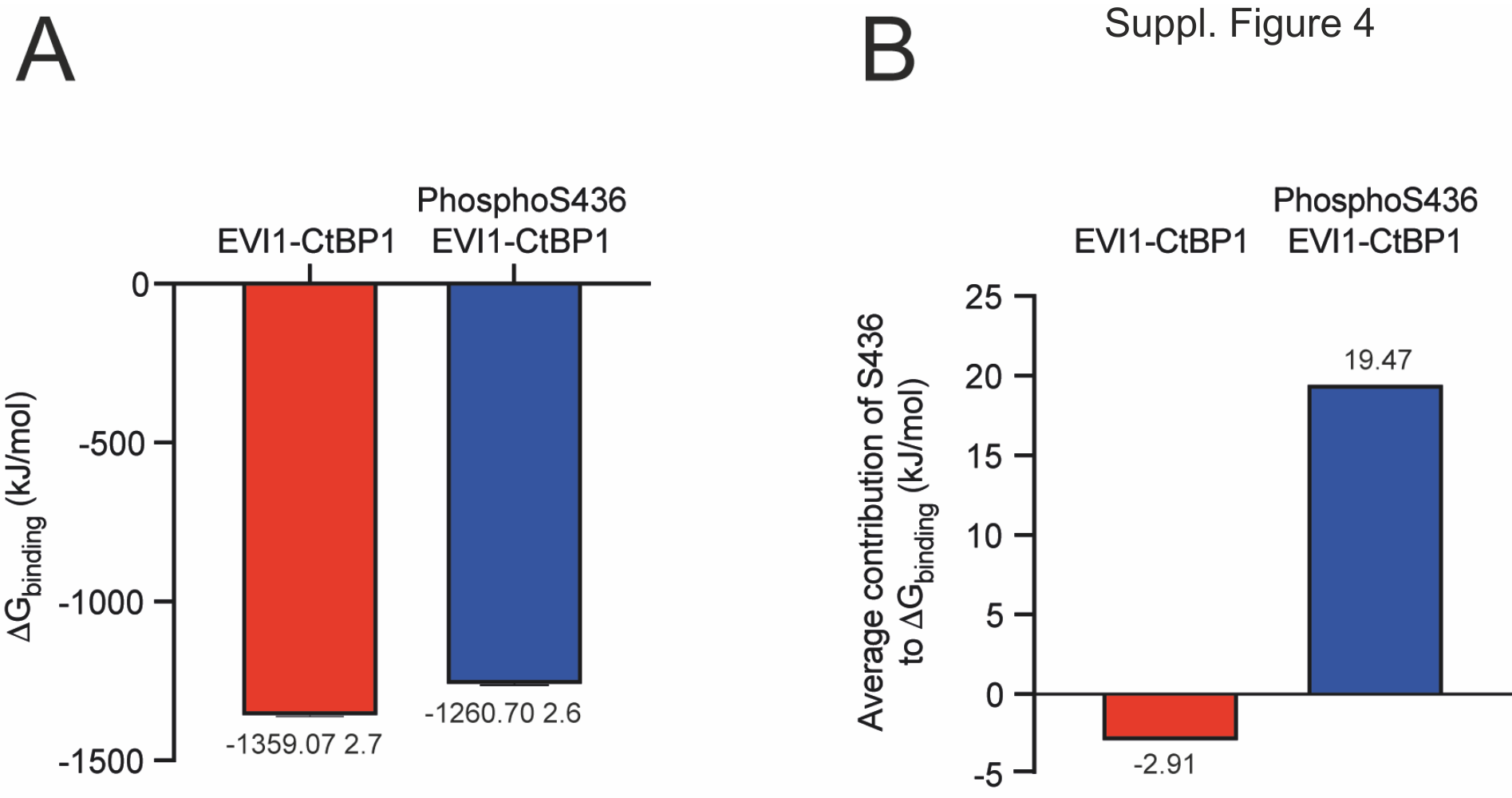

Supplement: Supplementary file 5 — Supplementary Figure 4 [file 41419_2020_3099_MOESM5_ESM.png]

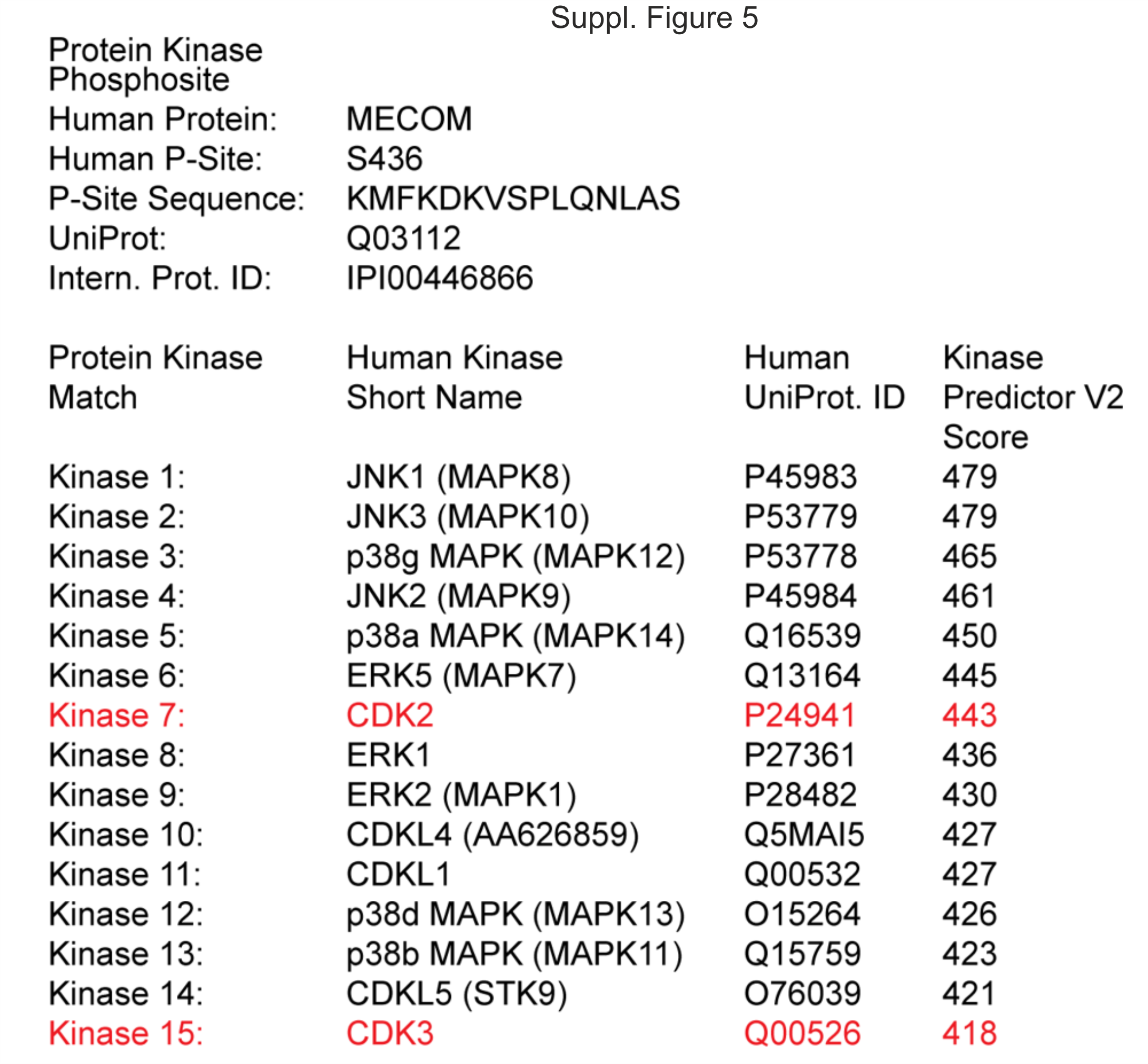

Supplement: Supplementary file 6 — Suppl. Figure 5 [file 41419_2020_3099_MOESM6_ESM.png]

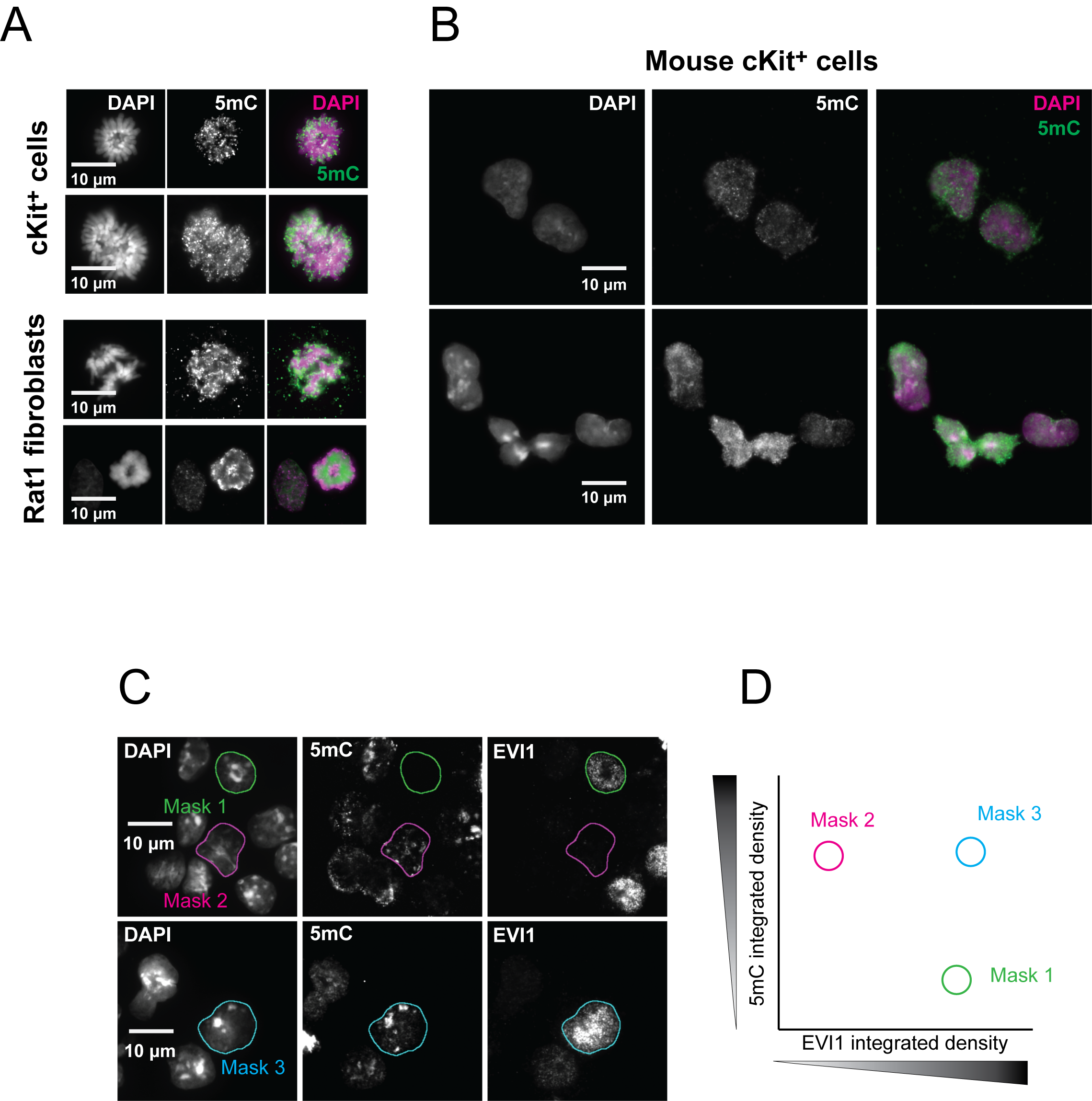

Supplement: Supplementary file 7 — Supplementary Figure 6 [file 41419_2020_3099_MOESM7_ESM.png]
